# Transcriptome Analysis of Alternative Splicing Events Induced by Arbuscular Mycorrhizal Fungi (*Rhizophagus irregularis*) in Pea (*Pisum sativum* L.) Roots

**DOI:** 10.3390/plants9121700

**Published:** 2020-12-03

**Authors:** Evgeny A. Zorin, Alexey M. Afonin, Olga A. Kulaeva, Emma S. Gribchenko, Oksana Y. Shtark, Vladimir A. Zhukov

**Affiliations:** All-Russia Research Institute for Agricultural Microbiology (ARRIAM), 196608 St. Petersburg, Russia; ezorin@arriam.ru (E.A.Z.); aafonin@arriam.ru (A.M.A.); okulaeva@arriam.ru (O.A.K.); egribchenko@arriam.ru (E.S.G.); oshtark@arriam.ru (O.Y.S.)

**Keywords:** transcriptomics, RNAseq, alternative splicing, arbuscular mycorrhiza, *P. sativum*

## Abstract

Alternative splicing (AS), a process that enables formation of different mRNA isoforms due to alternative ways of pre-mRNA processing, is one of the mechanisms for fine-tuning gene expression. Currently, the role of AS in symbioses formed by plants with soil microorganisms is not fully understood. In this work, a comprehensive analysis of the transcriptome of garden pea (*Pisum sativum* L.) roots in symbiosis with arbuscular mycorrhiza was performed using RNAseq and following bioinformatic analysis. AS profiles of mycorrhizal and control roots were highly similar, intron retention accounting for a large proportion of the observed AS types (67%). Using three different tools (SUPPA2, DRIMSeq and IsoformSwitchAnalyzeR), eight genes with AS events specific for mycorrhizal roots of pea were identified, among which four were annotated as encoding an apoptosis inhibitor protein, a serine/threonine-protein kinase, a dehydrodolichyl diphosphate synthase, and a pre-mRNA-splicing factor ATP-dependent RNA helicase DEAH1. In pea mycorrhizal roots, the isoforms of these four genes with preliminary stop codons leading to a truncated ORFs were up-regulated. Interestingly, two of these four genes demonstrating mycorrhiza-specific AS are related to the process of splicing, thus forming parts of the feedback loops involved in fine-tuning of gene expression during mycorrhization.

## 1. Introduction

Legume plants (family Fabaceae) are among the most symbiotically active groups of plants, since they are able to interact with a wide range of beneficial soil microorganisms [1,2]. In the course of evolution, legumes acquired the unique ability to develop two types of mutualistic endosymbioses: nitrogen-fixing symbiosis (NFS) with nodule bacteria and arbuscular mycorrhiza (AM), an ancient symbiosis with fungi of the division Glomeromycota [3].

The formation of symbiotic relationships between a legume plant and AM fungi or nodule bacteria is controlled by a large number of genes encoding receptors, components of the signal cascade, various transcription factors, and transporters [4]. Using the “forward genetics” approach, i.e., analysis of plant mutants, a large fraction of the genes participating in this process has been discovered [5,6]. The application of the “reverse genetics” approach to the study of symbiosis in legume plants led to the identification of hundreds of genes (so called “symbiosins”) that are specifically expressed in plant roots during the development of symbiosis [7]. Recent advances in “omics” technologies, namely, transcriptomics and proteomics, and combinations of these approaches made more comprehensive descriptions of molecular mechanisms underlying the development and functioning of nitrogen-fixing and AM symbioses in legumes possible [6,8].

The use of transcriptome analysis let the researchers to identify conservative genetic programs implemented during the development of NFS and AM [9] and individual components characteristic to the development of each symbiosis [10,11]. However, many details of the gene expression regulation in symbioses are still to be elucidated. As in many other biological systems, the expression of genes in symbiosis can be “fine-tuned” at various levels and in a variety of ways. An example of this principle is the regulation of the number of transcripts at the post-transcriptional level by means of alternative splicing (AS).

Alternative splicing (AS) is a mechanism that allows an individual gene to increase its coding capacity by producing several structurally different isoforms [12]. Most of the eukaryotic genes have an interrupted structure, that is, the alternation of exons (containing protein-coding sequences) and introns (the sequences that are subject to excision during splicing) [13]. This gene structure enables differential usage of some exons and introns, which creates a potential for production of protein isoforms with different functions.

Depending on the mechanism and the final exon-intron structure of the transcript, several types of AS are distinguished: exon skipping (SE), intron retention (RI), alternative donor site (A5), and alternative acceptor (A3) [14,15].

Alternative splicing is a powerful tool for regulating gene expression and, as a consequence, the amount of mRNA and protein in a cell [16]. One possible consequence of AS is nonsense-mediated decay (NMD) caused by an inclusion of a premature stop codon in coding sequence. The NMD system identifies aberrant mRNAs with a premature stop codon and selects them for further elimination, thereby preventing the accumulation of potentially harmful and dangerous proteins, or an alternatively spliced isoforms of a regulated gene [17].

Alternative splicing is an intrinsic part of plant responses to biotic and abiotic stress, influencing the quantitative ratio of transcripts in the cell and thus gene expression [18,19,20]. The interaction of plants with microorganisms (both pathogenic and mutualistic) includes modulation of stress reactions, AS events should be observed during NFS and AM development. However, so far, only four genes have been described in detail as undergoing AS events during NFS formation in legumes: *SIP1* [21], *HAP2-1* [22], *SYP132* [23,24], *DNF2* [25]. For AM symbiosis, the only described legume gene with AS events detected during mycorrhization is *SYP132* of *Medicago truncatula* Gaertn. [23] The studies on AS in garden pea are limited too [26,27]. Thus, this work was aimed at characterizing the AS events during formation of AM in garden pea roots and identifying the genes that have isoforms specific to AM symbiosis, by analysis of RNAseq data obtained for mycorrhizal roots of *Pisum sativum* L.

## 2. Results

### 2.1. Bioinformatics Analysis of RNA Sequencing Data

The trimmed reads of pea mycorrhizal and non-mycorrhizal roots of pea cv. Frisson were mapped to the reference genome of pea cv. Caméor [28], over 90% of the reads in each sample were uniquely mapped. The mapping statistics are presented in Table 1.

**Figure 1:**
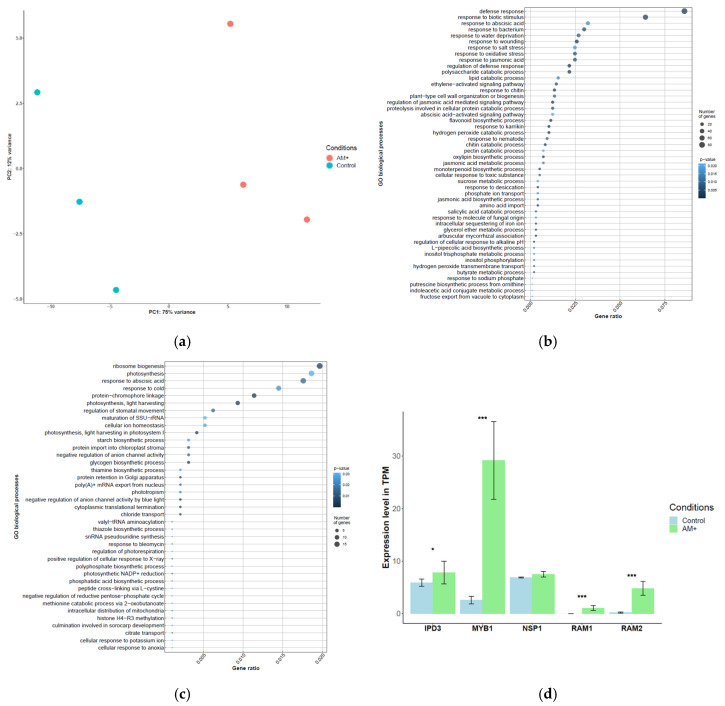
(**a**) Estimation of variance in RNA sequencing data by Principal Component Analysis (PCA). (**b**) Overrepresented GO “Biological process” of up-regulated genes in response to mycorrhization. (**c**) Overrepresented GO “Biological process” of down-regulated genes in response to mycorrhization. (**d**) Evaluation of the expression level of marker genes associated with arbuscular-mycorrhizal symbiosis in mycorrhizal and control roots of *P. sativum*. *—*p*-value < 0.05, ***—*p*-value < 0.001.

Principal component analysis (PCA) was used to describe the data, assess the variance among the samples and determine the influence of experimental factors on gene expression. Figure 1a shows that the first two components explain 87% of the variance, of which most (75%) is explained by component 1, which describes the influence of inoculation of the plants with AM fungi.

### 2.2. Detection of Differentially Expressed Genes in Response to AM Fungi Inoculation

In order to assess the plant’s response to mycorrhization, differential expression analysis was carried out to identify differentially expressed genes in samples inoculated with the AM fungi (versus control). It was found that out of 24,109 genes expressed in both control and mycorrhizal roots, the expression level of 685 genes was significantly increased, and 282 genes were significantly down-regulated in AM roots in comparison with control (non-colonized) roots (adjusted *p*-value < 0.05) (Supplementary Materials Table S1). Among the genes that were up-regulated in response to mycorrhization, a large group was associated with plant defense systems and responses to biotic stimuli, in particular, of bacterial origin. As expected, the expression levels of genes associated with responses to chitin and with flavonoid biosynthesis were also increased. Moreover, the genes directly related to the interaction of the plant with fungi and the functioning of arbuscules formed during the symbiosis between the pea and AM fungi have been identified (Figure 1b and Table S1). Genes associated with such common biological processes as sugar and starch biosynthesis, response to abscisic acid, ionic homeostasis, and photosynthesis were down-regulated in response to AM inoculation (Figure 1c, Tables S1 and S2). In order to further validate the establishment of an association between the plant and AM fungi, five known mycorrhization marker genes (*IPD3* [29], *MYB1* [30], *NSP1* [31], *RAM1* [32], *RAM2* [32]) were selected for the analysis of expression: as can be seen in Figure 1d, all genes, except for *NSP1*, were up-regulated in the roots inoculated with AM which indicates an intense plant response to inoculation and is a direct evidence of the formation of AM symbiosis in the experiment. The expression of phosphate starvation genes was assessed in order to see the effect of mycorrhization. It was found that of 9 tested genes only *PHO1* encoding a phosphate transporter with signaling function [33] showed up-regulation in mycorrhizal roots (Table S3).

### 2.3. Description of the Landscape of Alternative Splicing in the Control and Mycorrhizal Roots in P. sativum

Of the 24,109 genes expressed in the samples, 6026 undergo AS (i.e., have more than 1 isoform per gene). The algorithms of the SUPPA2 make it possible to identify the most common types of alternative splicing in the samples under study.

**Figure 2:**
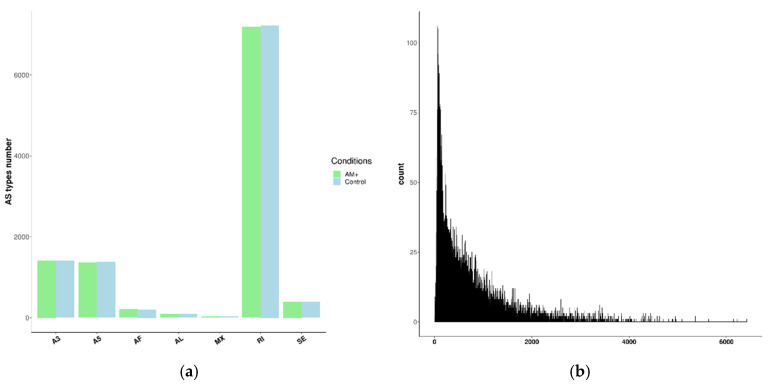
(**a**) Distribution of AS types in mycorrhizal and control roots of *P. sativum* (A3—alternative 3′ splice sites; A5—alternative 3′ splice sites; AF—Alternative First Exon; AL—Alternative Last Exon; MX—Mutually Exclusive Exons; RI—Retained Intron; SE—Skipping Exon); (**b**) Distribution of lengths of introns based on RNAseq data.

Thus, it was found that the most common type of AS in the roots of *P. sativum* is “intron retention” (67% of the total in both groups of samples) followed by alternative 3′ and 5′ splice sites. The average intron length is 618.3 bp, the median intron length is 422 bp (Figure 2b). These data are consistent with the previously shown distribution of AS types in plants [12,34,35,36]. At the same time, there is no noticeable difference in the distribution and number of types of AS (Figure 2a) and usage frequency of splice-site (Table 2) in the control and mycorrhizal roots.

### 2.4. Identification and Analysis of Differential AS Events in Mycorrhizal Roots

**Figure 3:**
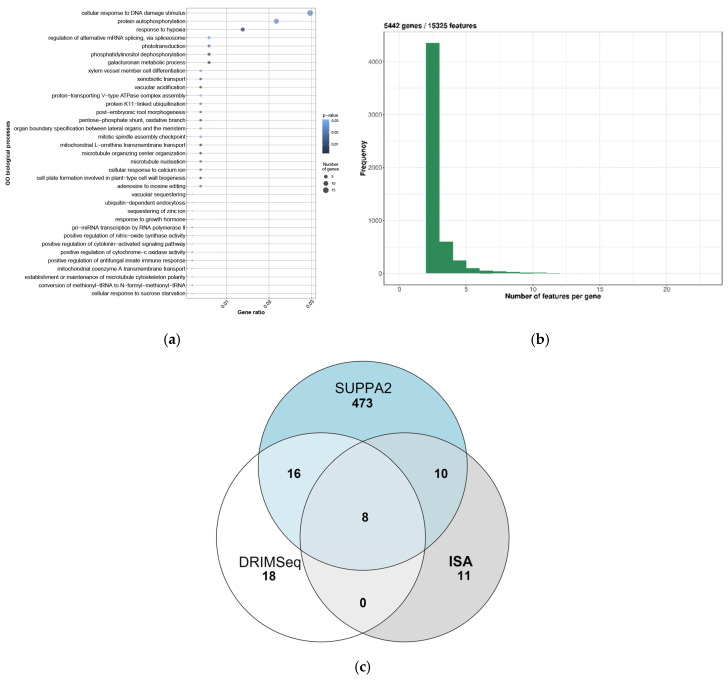
(**a**) Functional groups of genes demonstrating differential AS in mycorrhizal roots of *P. sativum;* (**b**) Distribution of the number of isoforms per gene in the analysis of AS using DRIMSeq and IsoformSwitchAnalyzeR; (**c**) Intersection of genes demonstrated the differential AS events obtained by three methods: SUPPA2, DRIMSeq, IsoformSwitchAnalyseR (ISA).

According to the results of SUPPA2, a total of 506 genes demonstrate differential AS events in mycorrhizal roots as compared to control (adjusted *p*-value = 0.05) (Table S4). Most of the genes belong to the “Cellular response to DNA damage stimulus”, “Protein autophosphorylation” and “Response to hypoxia” functional groups. (Figure 3a).

To analyze the AS using stricter parameters, the approaches implemented in the DRIMSeq and IsoformSwitchAnalyzeR packages were used. The criterion for filtering genes by expression level was applied: a gene was included in the analysis if it had more than one isoform, and its expression was detected in all replicates of one group of samples at a level of at least 10 reads per sample. As a result, we obtained 5442 genes with an average of 2.8 isoforms per gene (Figure 3b).

According to the results of DRIMSeq, 42 genes showed differential AS in mycorrhizal roots (Table S5). Among them, genes belonging to the functional groups “Histone methylation”, “Plant cell wall biogenesis”, “Signal transduction through phosphorylation” and “Response to molecules of fungal origin” were identified. According to IsoformSwitchAnalyzeR analysis, 29 genes were differentially spliced in mycorrhizal roots (Table S6). Among them were genes classified as “RNA processing”, “Defense response to bacteria”, and “Methylation”. All three algorithms used in the work showed the prevalent type of AS to be intron retention with the inclusion of a premature stop codon in the coding sequence of the transcript.

The coherence of the results obtained by different methods for detecting AS in RNA sequencing data appeared to be rather low: in our case, only eight genes were reliably identified by all the three algorithms, 16 were common when analyzed using DRIMSeq and SUPPA2, and 10 genes were common when analyzed by SUPPA2 and IsoformSwitchAnalyzeR (Figure 3c). Interestingly, the gene SYP132, the orthologue of which in *M. truncatula* has an AM-specific isoform expression level [23], was not identified by any of the programs used. We found *SYP132* (*Psat5g264800*) in our dataset and identified two isoforms of this gene in both control and mycorrhizal roots. However, the difference in their expression level between the control and AM roots was not statistically significant (*p*-value 0.00429, adjusted *p*-value 0.0785) (Figure S1).

For further analysis, genes showing differential splicing were selected according to the results of at least two of the three algorithms used in the work. In total 32 genes remained in the work after filtering (Table S7), among those, the most represented functional group was “developmental process” (eight genes). The remaining genes belonged to “response to molecule of fungal origin”, “cell tip growth”, “ATP-dependent chromatin remodeling”, “starch catabolic process”, and “positive regulation of endocytosis” groups. Most isoforms of these genes are generated by intron retention (27 genes), A3 and A5 types were detected for 11 and five genes, respectively, and finally, AS isoforms were produced by SE, AF and AL for five, two and one genes, respectively. For homologs of these genes, we counted the number of known isoforms in *M. truncatula* (which forms AM) and *Arabidopsis thaliana* (L.) Heynh. (which cannot form AM) using MtGEA, NCBI and TAIR services, and found that in *A. thaliana* only for 11 genes (34%) multiple isoforms were detected, whereas in *M. truncatula*, the isoforms were detected for 23 genes (72%). For nine genes, AS events were specific to pea.

**Figure 4:**
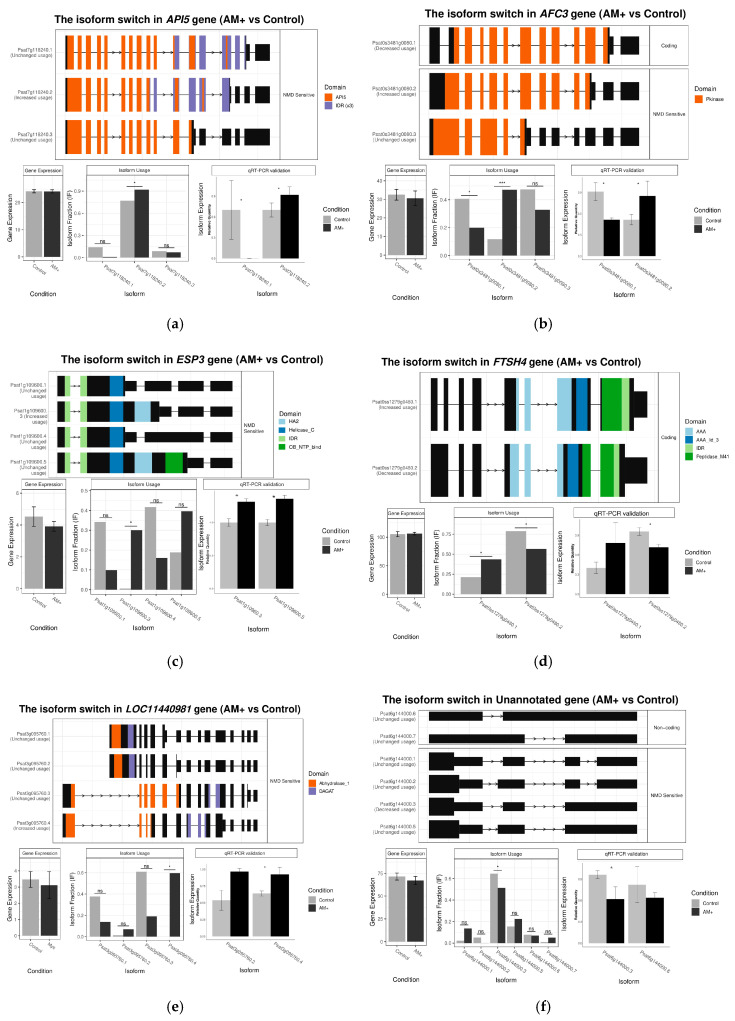

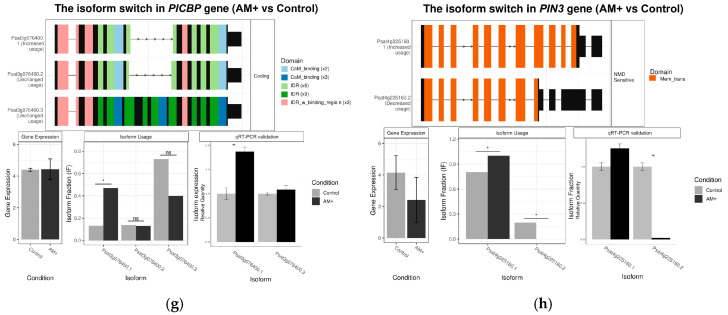
Structural and expression analysis of genes for which differential AS has biological consequences. (**a**) *API5* (*M. truncatula* genome ID *MTR_4g087935*; *P. sativum* genome ID: *Psat7g118240*); (**b**) *AFC3* (*M. truncatula* genome ID: *MTR_3g109390*; *P. sativum* genome ID: *Psat0s3481g0080*); (**c**) *ESP3* (*M. truncatula* genome ID: *MTR_6g061840*; *P. sativum* genome ID: *Psat1g109600*); (**d**) *FTSH* (*M. truncatula* genome ID: *MTR_5g075360*; *P. sativum* genome ID: *Psat0ss1279g0480*); (**e**) *LOC11440981* (*M. truncatula* genome ID: *MTR_7g083130*; *P. sativum* genome ID: *Psat3g095760*); (**f**) *Uncharacterized* (*P. sativum* genome ID: *Psat6g144000*); (**g**) *PICBP* (*M. truncatula* genome ID: *MTR_7g090500*; *P. sativum* genome ID: *Psat3g076400*); (**h**) *PIN3-like* (*M. truncatula* genome ID: *MTR_8g006780*; *P. sativum* genome ID: *Psat4g225160*); *—*p*-value < 0.05, ***—*p*-value < 0.001, ns—no significant difference.

Eight genes found by all three tools (Table S7), as most probable AS targets, were investigated further. These genes were: *API5* gene (the homolog of *MTR_4g087935*) encoding an apoptosis inhibitor protein, *AFC3* gene (the homolog of *MTR_3g109390*) encoding serine/threonine-protein kinase, *ESP3* gene (the homolog of *MTR_6g061840*) encoding pre-mRNA-splicing factor ATP-dependent RNA helicase DEAH1, *NUS1* gene (the homolog of *MTR_5g007260*) encoding dehydrodolichyl diphosphate synthase protein and *FTSH4* gene (the homolog of *MTR_5g075360*) encoding ATP-dependent zinc metallopeptidase. The functional annotation of the remaining three genes is unknown or questionable. All five annotated genes showed AS events generating isoforms with retained introns that contain a premature stop codon in the ORF, which probably leads to formation of truncated proteins and/or cleavage of the transcripts due to nonsense-mediated mRNA decay (Figure 4a–c,e). The ratio of the transcripts with premature stop codon is increased in mycorrhizal roots as compared to the control roots.

Finally, in order to validate the accuracy of the high throughput RNA-seq data, eight most reliable genes and two genes with high ratios between isoform content in control and mycorrhizal roots were selected, and the expression levels of the differentially expressed isoforms were estimated using qRT-PCR. These genes are: six out of eight highly reliable genes (no qRT-PCR was performed for *PUF* and *NUS1* due to peculiarities of sequences) as well as *PICBP* (the homolog of *MTR_7g090500*) encoding a calmodulin-binding protein and *PIN3-like* (the homolog of *MTR_8g006780*) encoding a component of the auxin efflux carrier. For these genes, qRT-PCR results were consistent with transcriptomics data (Figure 4). In the *PICPB* gene, AS leads to production of transcripts encoding either two or three calmodulin-binding domains (CaMBDs) (Figure 4g), and, according to both transcriptome and qRT-PCR data, the isoform with two CaMBDs is up-regulated in mycorrhizal roots in comparison with control (Figure 4g). The number of full-length transcripts of *PIN3-like* gene is increased in mycorrhizal roots and the number of the isoform with premature stop codon is, in contrast, significantly decreased (Figure 4h). The isoform of the *ESP3* gene with a premature stop codon and, consequently, without a nucleotide-binding domain, is significantly up-regulated in mycorrhizal roots according to both qRT-PCR and transcriptomics data (Figure 4c).

## 3. Discussion

To date, little is known about the role of alternative splicing in the systems formed by legume plants with symbiotic microorganisms. For the NFS, several examples of genes that underwent AS during nodule formation have been described in detail in model legumes *M. truncatula* and *Lotus japonicus* (Regel.) K. Larsen [21,22,23,24,25], while for the AM symbiosis, the information about the involvement of AS in its development and functioning in legumes is mostly lacking. AS is studied even more poorly in non-model objects.

The garden pea, an important crop plant and the oldest model in genetic studies, remains an attractive object for investigation into the molecular basis for the development and maintenance of symbioses due to the possibility to improve the symbiotic performance of pea cultivars. Recent advances in pea genome sequencing [28,37] enabled deep analysis of transcriptomes and proteomes of several pea genotypes in diverse conditions and, in particular, made it possible to analyze features of gene expression regulation such as alternative splicing events. In our earlier work we described for the first time several cases of AS, which were specific for nitrogen-fixing nodules of pea [38]. The results allowed us to conclude that specific ratios of isoforms of some genes (*PsPR10*, *PsWRKY40*) are observed in nitrogen-fixing nodules and that, in general, AS is more intensively carried out in nodules than with root tips. In the present study, we aimed to describe the differences in the landscapes of alternative splicing in control and mycorrhizal roots and to identify AS events specific to mycorrhizal roots of pea.

We showed that the AS landscape in both control and mycorrhizal roots is highly similar. The most common type of AS in pea roots, as in plants in general, was shown to be intron retention, which is consistent with literature data [12,34,35,36]. Mycorrhization didn’t influence the frequencies of AS types significantly, so the usage of different AS types was common both in control and mycorrhizal roots. At the same time, various in silico analysis methods for detecting and analyzing AS events showed significantly dissimilar results, due to the difference in the mathematical models implemented in them and the parameters used in these models. For this reason, it was decided to focus on the genes, which reliably demonstrate AS events according to at least two algorithms, and for which the structural changes in the mRNA sequence may have biological consequences as a result of the functional changes at the protein level. However, our criteria may have been too strict, since the gene *SYP132*, orthologue of which proved to have AM-specific isoform expression in *M. truncatula*, was not identified in our data as having differentially expressed isoforms after multiple testing correction. On the other hand, the molecular mechanisms of AM development and the role of AS events in particular genes may be slightly different between pea and *M. truncatula*.

Thus, in this work, 32 genes were identified that satisfy the chosen criteria and only eight genes can be considered as high-confidence, since they were identified by all three programs used. For some genes, the biological consequences of AM-specific alternative splicing events can be deduced. The *PICBP* gene encodes a calmodulin-binding protein, which is known to be involved in the regulation of responses to biotic stress and defense responses in *A. thaliana* [39,40,41]. During the processing of the pre-mRNA of this gene, two distinct isoforms are generated: the variant which has two calmodulin-binding domains (CaMBDs), and the isoform with retained intron where the third CaMBD is encoded (Figure 4d). According to our data, the canonical variant with two CaMBDs is significantly up-regulated in mycorrhizal roots (and the isoform with three CaMBDs is down-regulated, not statistically significant). Since the number of CaMBDs should affect the calmodulin binding capacity of the protein, the up-regulation of this particular isoform in mycorrhizal roots may be related to the modulation of the defense response via suppression of calmodulin-dependent signaling pathways.

In plants, many transcript isoforms are produced as a result of intron retention, which leads to the inclusion of a premature stop codon in the coding sequence. Such isoforms often become targets for the nonsense-mediated decay (NMD) system, which subjects transcripts containing a stop codon far from the polyA tail to degradation in order to prevent the accumulation the defective and potentially harmful proteins [17,35,42]. The process of plant roots colonization by the AM fungus does not stop after initial infection, and secondary infection events are triggered after the first stages of root colonization. It is known that various gene families, in particular those associated with the functioning of the cell wall, membrane, the organization of the cytosol, the cell cycle and initiation of apoptosis, are differentially regulated during the formation of this type of symbiosis [43]. Therefore, the suppression of the expression of genes associated with these processes, in particular, by means of AS followed by the involvement of the NMD system, seems appropriate in this context. This is the case for the *API5* gene encoding an apoptosis inhibitor protein (Figure 4a): two isoforms of this gene having a retained intron with a premature stop codon (which are, according to the predictions of the ISA tool, are targets for the NMD system) are expressed in mycorrhizal roots, while an isoform with the longest ORF is expressed only in non-colonized roots. We can hypothesize that this may be related to the suppression of apoptosis in non-inoculated roots and the removal of this blockage in the roots that establish an association with the AM fungi [43].

Phytohormones, such as auxin and abscisic acid (ABA), play an essential role in plant-microbe interactions [44,45]. Both perception and transport of auxin are involved in establishing and maintaining AM symbiosis [46,47]. PIN efflux proteins are the main regulators of polar auxin transport in the root apical zone [48]. We have shown that for one of them, PIN3-like, the expression in pea mycorrhizal roots can be regulated via alternative splicing (Figure 4g). This is consistent with the fact that the functioning of these proteins affects the success of the symbiosis through direct participation in auxin transport [49]. The differential splicing in pea mycorrhizal roots was recorded for the gene *CAR11* encoding the ABA-linked C2-domain containing protein (Table S6), which modulates the interaction of abscisic acid receptors with the plasma membrane and, thus, regulates the plant’s sensitivity to abscisic acid [50]. The full-length transcript of *CAR11* gene is significantly up-regulated in mycorrhizal roots, while the truncated or NMD-sensitive transcript is down-regulated. Since abscisic acid signaling promotes root colonization by the AM fungus [45,51], it can be assumed that the differential splicing of mRNA of this gene and increasing the amount of functional protein may lead to the modulation of the interaction of abscisic acid receptors with the membrane, ultimately increasing the sensitivity of the plant to abscisic acid thus facilitating the process of root colonization by the AM fungi.

The plant defense reactions can also be modulated via AS events. We found an intron retention leading to a premature stop codon in the ORF of the receptor-like serine/threonine protein kinase gene *CRK25* (Table S6). In this case, it is likely that the stop codon either leads to the synthesis of a protein with a truncated kinase domain or degradation of transcript via the NMD. In the first case isoform can continue to perform its function related to the binding of ligands (the signals probably related to the presence of the pathogenic microorganisms, in particular, fungi), but is not able to phosphorylate its targets, thus interrupting signal transduction associated with the plant’s defense systems. The differential splicing of the transcripts of this gene according to any of the suggested scenarios can rearrange the plant defense system in order to enter into association with nonpathogenic AM fungi.

Interestingly, among alternatively spliced transcripts, we found those involved in regulation of alternative splicing. This is the case for the *AFC3* gene (Figure 4b) that belongs to a serine/threonine-protein kinases gene family, members of which mediate expression of genes of several splice factors. Overexpression of AFC proteins leads to changes in the alternative splicing patterns of several genes, including those related to plant development by differential choice of the splice sites [52,53]. We found that the full-length transcript of *AFC3* is strongly down-regulated and isoform with PTC, in contrast, is increased, in the pea mycorrhizal roots. We can assume that this mechanism is used for the fine-tuning of gene expression during mycorrhization via influence on active concentration of AS factors that regulate AS. A similar case, probably, is illustrated by the AS of the *ESP3* gene (Figure 4c), which, as a splicing factor, is involved in the plant development (including the roots) [54]. In pea mycorrhizal roots, the increase of an alternatively spliced isoform with a premature stop codon (as a result of intron retention) can be associated with subsequent changes in the regulation of splicing processes in mycorrhizal roots.

Finally, the *M. truncatula* homologs of 23 genes out of 32 considered as demonstrating AS events specific for pea mycorrhizal roots showed the existence of similar isoforms. At the same time, in *A. thaliana*, a model plant which cannot form AM, only 11 homologs were found to have similar AS pattern (Table S7). This implies that the obtained isoforms in pea and *M. truncatula* may represent a common mechanism characteristic for several plant species, which are able to interact with AM fungi. On the other hand, the presence of genes having similar number of isoforms in all three tested plants may indicate that the pattern of splicing of these genes is conservative, but the mechanisms of individual isoforms expression regulation are rearranged in plants which are able to interact with AM. Thus, further work will contribute to full description of AS landscape in pea, considering common AS events and the ones specific for this object.

## 4. Materials and Methods

### 4.1. Plant Materials and Experimental Conditions

The seeds of the cv. Frisson were sterilized in concentrated sulfuric acid for 10 min, followed by three rinses with sterile water. Seeds were germinated for three days at 23 °C in the dark on Petri dishes containing sterile 1% agar-agar media. Pea seedlings were planted into 1 L microbox containers, which ensure sterility inside the box and free gas exchange (Sac O2, Deinze, Belgium, https://saco2.com/more-info-microbox/), filled with a growth substrate sterilized by autoclaving for 60 min at 134 °C and 0.22 MPa. The growth substrate consisted of a mixture (1:1 *v*/*v*) of quartz sand (fraction 0.6–0.8) and opoka-rock (silica rich marl), supplemented with 1 g·L^−1^ calcium orthophosphate and 0.35 L·L^−1^ 0.5× modified Hoagland’s solution without phosphate [55]. The containers were filled with the growth substrate up to 0.5 cm from the top and three pea seedlings were planted through specially made holes in the lid of the container so that the root was inside the container, while the shoot with the cotyledons remained outside. The cotyledons were fixed in the holes using plasticine, autoclaved for 30 min at 112 °C and 0.05 MPa.

The AM fungus *Rhizophagus irregularis* strain BEG144 provided by the International Bank for the Glomeromycota (Dijon, France) was used to inoculate pea seedlings. *Plectranthus australis* R. Br. was used as a host plant for *R. irregularis* cultivation. Fresh roots of *P. australis* colonized by *R. irregularis* were surface-disinfected as described by Cranenbrouck et al. [56], cut for 0.5–1 cm segments and used as AM fungal inoculum in this study (0.3 g of inoculum per plant). Half of the containers were supplemented with inoculum into wells made in the substrate before planting; the other half was left as uninoculated control.

Pea plants in microbox containers were grown in the growth chamber Vötsch VB 1514 (Vötsch Industrietechnik GmbH, Reiskirchen, Germany) under controlled conditions at 16/8 h and 21/19 °C day/night regime. The experiment was carried out in three biological replicates, 6 plants per replicate. Whole pea root systems were harvested after 28 days of cultivation and several lateral roots (15 cm length) from each pea root system were randomly selected and frozen at −20 °C and then subjected to analysis of AM development as described by Shtark et al. [55]. After that, the root systems were immediately frozen in liquid nitrogen and stored at −80 °C for subsequent RNA isolation. The parameters of mycorrhizal colonization were estimated according to Trouvelot et al. [57] and are indicated in Table S8.

### 4.2. RNA Extraction, cDNA Synthesis, qRT-PCR and Sequencing

The roots were ground using mortar and pestle in liquid nitrogen, RNA was isolated using Trizol (Thermo Fisher Scientific, Waltham, MA, USA) according to the manufacturer’s protocol with minor changes. The RNA quality was assessed visually using gel electrophoresis in 1.5% agarose gel, the concentration RNA was measured using a Qubit Fluorometer and Qubit RNA BR Assay Kit (Thermo Fisher Scientific), strand-specific RNAseq libraries preparation was performed using TruSeq Stranded mRNA LT Sample Prep Kit (Illumina, San Diego, CA, USA) and sequencing using Illumina technology (Macrogen, Seoul, Korea). The RNA-Seq data are publically available at the SRA database of NCBI (https://www.ncbi.nlm.nih.gov/) under project accession number PRJNA664581.

The expression patterns of 8 genes that undergone AS were analyzed using qRT-PCR. Two independent biological replicates of control and mycorrhizal root tissue were used for RNA extraction for qRT-PCR assays. The reverse transcription reactions were carried out by ThermoScientific RevertAid First Strand cDNA Syntesis kit (Thermo Fisher Scientific). Transcript-specific primers were designed and presented in Table S9. All reactions were performed on an CFX96 Touch Real-Time PCR Detection System (Bio-Rad, Hercules, CA, USA) with three technical replicates. Each reaction was performed in a total volume of 10 μL including 0.3 μM primer pairs, 1 μL diluted cDNA, and 2 μL 5× SYBR Green qPCRmix-HS SYBR (Evrogen, Moscow, Russia). The amplification reactions were incubated at 95 °C for 30 s, followed by 39 cycles of 95 °C for 5 s, 58 °C for 30 s, and 7 °C for 30 s. *P. sativum GapC1* gene was used as a normalizer gene. Relative gene expression levels were calculated using the 2^−∆∆*C*t^ method. Statistical analysis of the results was performed on deltaCT values using ANOVA.

### 4.3. Bioinformatic Analysis

The FastQC (version 0.11.8) was used to assess the quality of raw reads. Adapters, low quality reads, human and bacterial contaminants were removed using the BBDuk program (version 37.90) from the BBTools suite (https://sourceforge.net/projects/bbmap/). Paired-end reads were mapped to the reference genome of the cv. Caméor and the gene expression was measured using STAR (version 2.7.3a.) [58]. The mapping of reads to the genome was carried out in two steps. To determine the gene expression levels and to identify differentially expressed genes, mapping was performed in the STAR program with the—quantMode GeneCounts option. To quantify isoforms for subsequent analysis of alternative splicing, reads were mapped using STAR with the—quantMode TranscriptomeSAM option with subsequent quantification of isoforms in Salmon (version 1.2.1) [59].

Differential gene expression analysis was performed in the DESeq2 [60] package in the R environment. Analysis of alternative splicing at the level of local events was carried out using the SUPPA2 [15], and at the isoform level using the SUPPA2 program, as well as the DRIMSeq [61] and IsoformSwitchAnalyseR [62] packages in the R. In DRIMSeq, the results were filtered with an adjusted *p*-value of 0.1, and in SUPPA2 and the IsoformSwitchAnalyseR *p*-values remained default.

GO terms for genes of interest were obtained using the Trinotate suite [63]. GO enrichment analysis (using the weight01 algorithm and Fisher’s exact test) and further visualization were performed in the packages topGO [64] and ggplot2 [65], respectively.

The search for orthologous pea genes in the *M. truncatula* genome was performed via the web service https://mtgea.noble.org, and in *A. thaliana* genome—using blastn against ncbi non-redundant nucleotide database and Arabidopsis Information Resource (TAIR).

## 5. Conclusions

In this work a comprehensive analysis of alternative splicing in mycorrhizal roots of pea cv. Frisson, using RNA sequencing and bioinformatic methods was carried out for the first time. This made it possible to identify a set of genes whose differential spliced mRNA could potentially influence the formation and maintenance of AM symbiosis. It should be noted that, until recently, such studies were carried out only on model legumes *M. truncatula* and *L. japonicus*, however, the development and reduction in the cost of methods of high-throughput sequencing enables the extension of the ‘omics’ approaches to so called ‘orphan crops’, including the garden pea [66]. However, since the results of the study were obtained mainly with use of computational methods, the detailed functions of the identified differentially spliced transcripts and its participation in symbiotic system AM fungi—*P. sativum* should be verified by molecular biology methods in the future.

## Figures and Tables

**Table 1:** Information on the mapping of RNA sequencing reads on the genome of *P. sativum* cv. Caméor.

Sample Name	No. Reads before Trimming	No. Reads after Trimming	% Uniquely Mapped Reads	% of Reads Mapped to Multiple Loci
Control.1	21,904,071	21,700,136	92.70	2.01
Control.2	30,815,955	30,517,632	94.05	2.05
Control.3	25,026,957	24,787,916	94.72	2.07
AM.1	25,985,674	25,711,178	93.24	2.07
AM.2	25,226,401	24,959,410	93.39	2.11
AM.3	26,840,632	26,515,644	94.35	2.08

**Table 2:** Usage frequency of different splice-sites based on RNAseq reads mapping using STAR.

	GT/AG	GC/AG	AT/AC	Non-Canonical
Control	15,475,479	160,638	16,588	37,918
AM+	15,174,737	154,958	15,490	36,487

## Supplementary Materials

The following are available online at https://www.mdpi.com/2223-7747/9/12/1700/s1, Table S1: The results of Differential expression analysis; Table S2: GO terms for annotated DE genes; Table S3: Estimation of pea phosphate starvation response genes; Table S4: The list of all differentially spliced genes detected by SUPPA2; Table S5: The list of all differentially spliced genes detected by DRIMSeq; Table S6: The list of all differentially spliced genes detected by IsoformSwitchAnalyzeR; Table S7: table describing the final set of 32 genes demonstrating AS; Table S8: Table with estimation of root colonization by AM; Table S9: List of primer sequences used for qRT-PCR; Figure S1: Estimation of pea SYP132 gene isoforms expression in the analyzed samples.
