# Supplementary material for: Transcriptome Analysis of Alternative Splicing Events Induced by Arbuscular Mycorrhizal Fungi (Rhizophagus irregularis) in Pea (Pisum sativum L.) Roots
Source: Plants (Basel). 2020 Dec 3;9(12):1700. doi: 10.3390/plants9121700 (PMC7761762; doi:10.3390/plants9121700)
Supplement: Supplementary file 1 [file plants-09-01700-s001.zip › plants-937167 - figure s1 for XML.pdf]

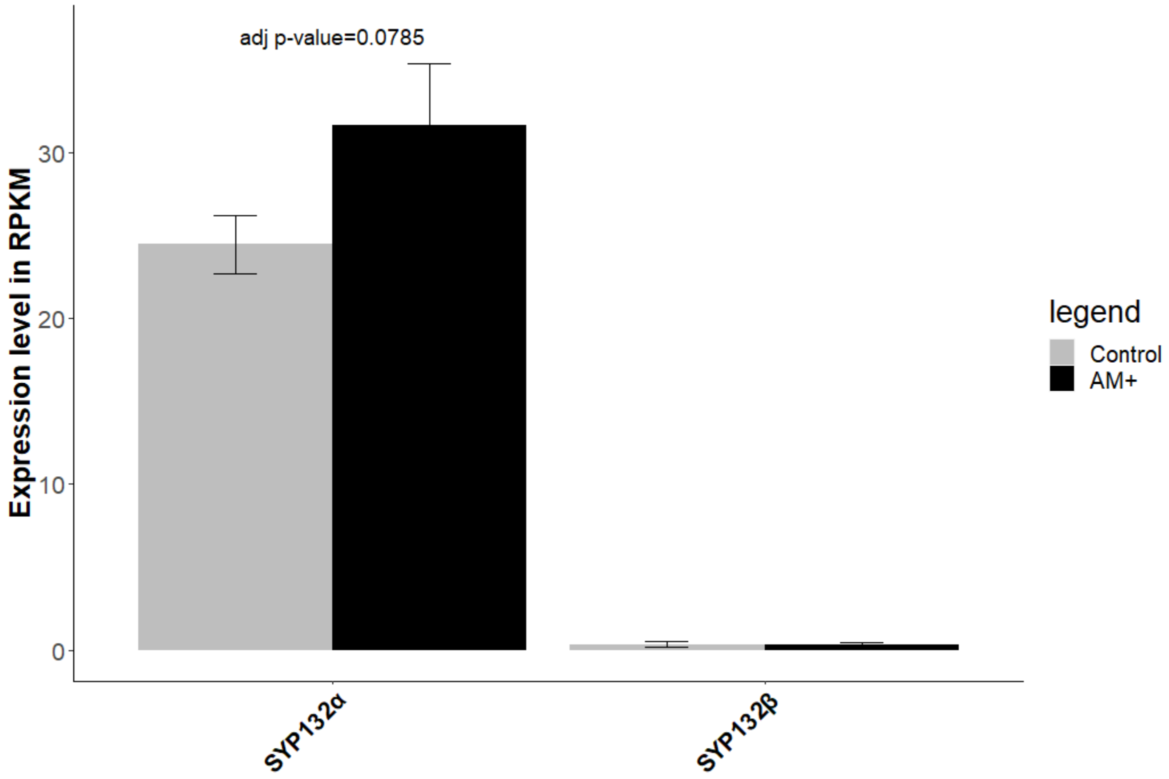

Figure S1. Isoforms expression analysis SYP132 gene in P. sativum. P-value was adjusted by Benjamini Hochberg correction method.
